# Key issues for stakeholder engagement in the development of health and healthcare guidelines

**DOI:** 10.1186/s40900-023-00433-6

**Published:** 2023-04-28

**Authors:** Jennifer Petkovic, Olivia Magwood, Lyubov Lytvyn, Joanne Khabsa, Thomas W. Concannon, Vivian Welch, Alex Todhunter-Brown, Marisha E. Palm, Elie A. Akl, Lawrence Mbuagbaw, Thurayya Arayssi, Marc T. Avey, Ana Marusic, Richard Morley, Michael Saginur, Nevilene Slingers, Ligia Texeira, Asma Ben Brahem, Soumyadeep Bhaumik, Imad Bou Akl, Sally Crowe, Laura Dormer, Comfort Ekanem, Eddy Lang, Behrang Kianzad, Tanja Kuchenmüller, Lorenzo Moja, Kevin Pottie, Holger Schünemann, Peter Tugwell

**Affiliations:** 1 Bruyère Research Institute, Ottawa, Canada; 2 Faculty of Medicine, University of Ottawa, Ottawa, Canada; 3 Interdisciplinary School of Health Sciences, University of Ottawa, Ottawa, Canada; 4 McMaster University, Hamilton, Canada; 5 Clinical Research Institute, American University of Beirut Medical Center, Beirut, Lebanon; 6 The RAND Corporation and Tufts University School of Medicine, Boston, MA USA; 7 School of Epidemiology and Public Health, University of Ottawa, Ottawa, Canada; 8 Nursing Midwifery and Allied Health Professions (NMAHP) Research Unit, Glasgow Caledonian University, Glasgow, UK; 9 Tufts Medical Center, Tufts Clinical and Translational Science Institute, Boston, MA USA; 10 Tufts Medical Center, Institute for Clinical Research and Health Policy Studies, Boston, MA USA; 11 Department of Internal Medicine, American University of Beirut, Beirut, Lebanon; 12 Department of Health Research Methods, Evidence, and Impact (HEI), McMaster University, Hamilton, ON Canada; 13 Department of Health Research Methods, Evidence and Impact, McMaster University, Hamilton, ON Canada; 14 Department of Anesthesia, McMaster University, Hamilton, ON Canada; 15 Department of Pediatrics, McMaster University, Hamilton, ON Canada; 16 Biostatistics Unit, Father Sean O’Sullivan Research Centre, St Joseph’s Healthcare, Hamilton, ON Canada; 17 Centre for Development of Best Practices in Health (CDBPH), Yaoundé Central Hospital, Yaoundé, Cameroon; 18 Division of Epidemiology and Biostatistics, Department of Global Health, Stellenbosch University, Cape Town, South Africa; 19 Weill Cornell Medicine –Qatar, Doha, Qatar; 20 Ministry of Health, Cross River State, Calabar, Nigeria; 21 Cumming School of Medicine, University of Calgary, Alberta Health Services, Calgary Zone, Canada; 22 Canadian Council on Animal Care, Ottawa, Canada; 23 Department of Research in Biomedicine and Health, Center for Evidence-Based Medicine, University of Split School of Medicine, Split, Croatia; 24 Cochrane, St Albans House, Haymarket, London, 57-59 UK; 25 Hôpital Montfort, Ottawa, ON Canada; 26 Becaris Publishing Limited, Royston, UK; 27 Crowe Associates, Thame, UK; 28 South African Medical Research Council, Cape Town, South Africa; 29 Centre for Homelessness Impact, London, UK; 30 Director Guidelines and Care Pathways, INEAS (National Authority for Assessment and Accreditation in Healthcare), Tunis, Tunisia; 31 Meta-Research and Evidence Synthesis Unit, The George Institute for Global Health, New Delhi, India; 32 Department of Internal Medicine, American University of Beirut, Beirut, Lebanon; 33 Center for Advanced Studies in Biomedical Innovation Law (CeBIL), Faculty of Law, Copenhagen University, Copenhagen, Denmark; 34 School of Public Health, Bielefeld University, Bielefeld, Germany; 35 Department of Biomedical Sciences for Health, University of Milan, Milan, Italy; 36 Departments of Family Medicine and Epidemiology and Biostatistics, Western University, London, Canada; 37 Department of Family Medicine, University of Ottawa, Ottawa, Canada; 38 Clinical Epidemiology and of Medicine, WHO Collaborating Centre for Infectious Diseases, Research Methods and Recommendations, Hamilton, Canada; 39 Department of Health Research Methods, Evidence, and Impact, Cochrane Canada and McMaster GRADE Centre, McMaster University, Hamilton, Canada; 40 Department of Medicine, Faculty of Medicine, University of Ottawa, Ottawa, Canada; 41 Clinical Epidemiology Program, Ottawa Hospital Research Institute, Ottawa, Canada; 42 School of Epidemiology and Public Health, Faculty of Medicine, University of Ottawa, Ottawa, Canada; 43 WHO Collaborating Centre for Knowledge Translation and Health Technology Assessment in Health Equity, Bruyère Research Institute, Ottawa, Canada

**Keywords:** Guideline development, Stakeholder engagement, Patient and public involvement

## Abstract

Established in 2015, the Multi-Stakeholder Engagement (MuSE) Consortium is an international network of over 120 individuals interested in stakeholder engagement in research and guidelines. The MuSE group is developing guidance for stakeholder engagement in the development of health and healthcare guideline development. The development of this guidance has included multiple meetings with stakeholders, including patients, payers/purchasers of health services, peer review editors, policymakers, program managers, providers, principal investigators, product makers, the public, and purchasers of health services and has identified a number of key issues. These include: (1) Definitions, roles, and settings (2) Stakeholder identification and selection (3) Levels of engagement, (4) Evaluation of engagement, (5) Documentation and transparency, and (6) Conflict of interest management. In this paper, we discuss these issues and our plan to develop guidance to facilitate stakeholder engagement in all stages of the development of health and healthcare guideline development.

## Abstract (summary)

A group of international researchers, patient partners, and other stakeholders are working together to create a checklist for when and how to involve stakeholders in health guideline development. Health guidelines include clinical practice guidelines, which your healthcare provider uses to determine treatments for health conditions. While working on this checklist, the team identified key issues to work on, including: (1) Definitions, roles, and settings (2) Stakeholder identification and selection (3) Levels of engagement, (4) Evaluation of engagement, (5) Documentation and transparency, and (6) Conflict of interest management. This paper describes each issue and how the team plans to produce guidance papers to address them.

## Introduction

In recent years, interest in stakeholder engagement in the development of health and healthcare guidelines has increased. This is demonstrated by the increasing number of tools being developed to assist guideline developers with involving certain groups in their guideline processes, particularly patients and members of the public [1, 2]. We define a stakeholder as people and groups who are responsible for or affected by health and healthcare-related decisions [3]. We recognize that this term is problematic for some populations and are actively working with relevant groups to select and suitable alternative term (https://theoche.ca/f/muse-work-and-terminology).

By ‘engagement’, we mean the approach to gather input or contributions from stakeholders resulting in “informed decision-making about the selection, conduct, and use of the research” [8].

In addition to patients and the public, there are other stakeholder groups who are responsible for or affected by health and healthcare-related decisions, such as payers/purchasers of health services, peer review editors, policymakers, program managers, providers, principal investigators, product makers, and payers of health research. Appropriate participation by each of these groups should be considered through discussions amongst the groups themselves in addition to engagement with the guideline development panel [4–6]. This ensures that feedback from each group is shared with the other groups which may help improve the guideline and its relevance.

In 2015, research teams from Canada, the US, and the UK met virtually to discuss mutual interests in improving stakeholder engagement in research and guidelines. The group identified gaps in guidance related to how to engage different groups in a meaningful way across all types of research and guideline development. To address these gaps, we established the Multi-Stakeholder Engagement (MuSE) Consortium which has grown to become an international network of over 120 individuals from 20 countries. All members have an interest and expertise in different aspects relevant to stakeholder engagement in research. The group is governed by a core team who manage the daily tasks of the group as well as stakeholder group co-leads. Members of the consortium are invited by email and newsletters to contribute to projects or tasks if they are interested and can use the network to share related work.

We are currently developing guidance for multi-stakeholder engagement in the development of health and healthcare guidelines. To inform the production of this guidance, we held a number of exploratory discussions with members of the MuSE Consortium and individuals representing our identified stakeholder groups. The aim of this paper is to report and discuss key issues for multi-stakeholder engagement in guideline development.

## Methods

The stakeholder groups identified within our protocol, based on published work [3, 7, 8], include patients, payers of health research, payers of health services, peer review editors, policymakers, principal investigators and members of the research team, providers, product makers, program managers, and members of the public (See Table 1).

**Table 1:** Stakeholder categories and their descriptions with examples

Stakeholder category	Description	Examples
Patients, patient caregivers, patient advocates/ organizations	Those with lived experience with the condition of interest or who care for or advocate on behalf of those with lived experience	- Patient with condition on which the guideline is focused - Caregiver of patient on which the guideline is focused - Patient group representing patients on which the guideline is focused
Payers of health research	Individuals and organizations that fund research projects, such as government funders, industry funders, foundations	- National health research funding agency - Philanthropic foundations
Payers / Purchasers of Health Services	Individuals, organizations and entities that pay for health services	- Public health systems - Private insurers
Peer Reviewed Journals Editors	Those who set journal policy on guidelines and manage the peer review process and editing	- Editor or editor-in-chief of academic journal
Policymakers	Individuals, organizations and entities that craft public or private policy (on health) at any level of government	- Regulators - Health ministries
Principal Investigators and all members of the of research team	Individuals, organizations, and associations that conduct or advocate health research	- Trialist - Systematic reviewer - Study manager
Product makers	Individuals working for companies that manufacture pharmaceuticals, medical devices, medical procedures, health technologies, or for profit educational and behavioral packages	- Pharmaceutical company - Medical device manufacturer - Digital / Guided programs available for purchase (e.g. cognitive behavioral therapy programs)
Program managers	Managers/directors who plan, lead, oversee, or deliver any program that provides public health, community services, or clinical care (e.g. budgeting, hiring, staffing, organizing, coordinating, reporting)	- May be health care providers but are not on the front-line delivering health care related to the program of interest, such as a person overseeing an immunization program but not delivering vaccinations
Providers	Persons and their professional associations who provide health care in a professional capacity and allowed by regulatory bodies to provide a health care service	- Psychologists, clinical pharmacists, nurses, physiotherapists, dentists, optometrists, physician assistants, physicians, optometrists, physician assistants and physicians
Public	Individuals in the general population of a defined geographic area excluding patients, caregivers, and health professionals living or working with the condition of interest	- Member of the public with no specific experience with the intervention or condition on which the guideline is focused

We recruited 2–4 co-leads for each identified stakeholder group. Co-leads were selected for their specific, recognized expertise relevant to their stakeholder group (for example, lived experience or research experience). These individuals were identified through snowballing starting with suggested contacts from our core team and the suggestions of those who were contacted. We aimed to ensure co-leads were balanced between high- and low- and middle-income countries.

The objective of this project is to develop a Stakeholder Engagement Checklist Extension of the GIN-McMaster Guideline Development Checklist [9], to be used to gain broad feedback from relevant stakeholders. In February 2021, we held a 3-day virtual meeting with all of our stakeholder group co-leads and other members of the MuSE Guidelines project team. Several cross-cutting issues were identified which warrant further consideration as the Stakeholder Engagement Checklist Extension is developed.

The co-leads for each stakeholder group and the complete meeting participant list are presented in Appendix 1 and our GRIPP2 checklist is in Appendix 2.

## Results

Six substantive issues were identified as being important for further exploration.

These were:

1. Definitions, roles and setting
2. Stakeholder identification and selection
3. Levels of engagement
4. Evaluation of engagement
5. Documentation and transparency
6. Conflict of interest management

A description of key considerations for stakeholder engagement in health and healthcare guideline develop is provided below.

### Definitions, roles and setting

Stakeholders may include not only those directly involved in guideline development but also those involved in preparing research, conducting research, sharing evidence, or implementing that evidence.

Our team has previously reviewed a number of frameworks for categorizing stakeholder groups in research [3, 10]. There are many similarities, and several differences related to preferences for lumping, splitting, refining the groups or tailoring to optimize for the specific context of guidelines. For example, in some research settings patients and the public are combined into one stakeholder group, but for the MuSE guidelines projects our public and patient stakeholders asserted that these two groups had different perspectives and would be interested in engaging in different aspects [11] of the guideline development process, therefore should be considered separately.

Similarly, our stakeholders agreed that we should separate peer-reviewed journal editors from principal investigators and the research team. Academic journals often have requirements for the methods, rigor, and format of publishable guidelines which will require different input than the research team. The research team may be contributing from the perspective of those who have conducted primary research or as systematic reviewers who have assessed the body of evidence on a topic requiring engagement in different aspects of guideline development.

Where other frameworks have separated payers and purchasers of health services into two distinct groups, we have lumped these together because there are many health care systems in different countries that do not make this distinction and therefore their role for guideline development would be the same.

We suggest that there are 10 stakeholder groups that warrant individual attention for health and healthcare guideline development. The roles that each of these groups play, whether providing feedback or contributing to decision-making, may depend on the guideline and its setting. We recognize that the list provided in Table 1 is not exhaustive and there may be other important stakeholders for guideline development, depending on the context and setting.

Our activities prompted considerable debate on whether the organizations that commission guidelines and the guideline secretariats, which we call Px, are stakeholders that meet our definition of interested people and groups. Although we agree that the Px group are key actors and their interactions with the stakeholder groups are essential, we have decided to keep them separate from our 10 stakeholder groups for two reasons. First, this group likely has a decision-making role throughout all stages of guideline development. Second, the guidance that we intend to develop is aimed at how the Px group should engage with other important stakeholder groups. Therefore, the decisions made by the Px group are guided by other documents, such as organizational handbooks, and our guidance is intended to assist the Px with engaging other groups.

### Stakeholder identification and selection

The selection of specific individuals to represent each group requires consideration. To address these challenges around identification, we have developed a list of factors to consider when selecting individuals to engage in health research (see Box 1) [12].

**Box 1:** Factors to consider during the identification and invitation of individuals for research partnership [12].

*Highly desirable* 1. Ability and willingness to represent stakeholder group 2. Commitment and time capacity 3. Communication skills 4. Financial and non-financial relationships and activities, and conflicts of interest 5. Expertise or experience 6. Inclusivity (equity, diversity, and intersectionality) 7. Training, support, and funding needs *Desirable* 8. Influence 9. Previous stakeholder engagement 10. Research relevant values	

Clarity is needed when one individual can represent more than one stakeholder group, for example a policymaker who is also a patient, or a principal investigator who is also a provider. A ‘positionality statement’, which allows each member of the guideline panel to declare which stakeholder group(s) they are representing in addition to the groups they could have represented, may be helpful [13]. This could be developed by each individual with guidance from the guideline secretariat to ensure that the roles are filled as needed for the specific guideline.

### Levels of engagement

The level of engagement of stakeholders in guideline development can vary. Previous stakeholder engagement work identified 4 levels of engagement adapted from other sources [14–16]: (1) Communication: Stakeholders receive information but have no role in contributing, (2) Consultation: Stakeholders provide their views, thoughts, feedback, opinions or experiences but without a commitment from the guideline developers to act on them, (3) Collaboration: Stakeholders are engaged to influence the production of the guideline (e.g. commenting, advising, ranking, voting, prioritizing, and reaching consensus) without direct control over decisions, and (4) Coproduction: Stakeholders are equal members of the guideline development team and have a key role in decision-making in the guideline development process. However, our experience assessing papers reporting on stakeholder engagement in guidelines identified challenges in operationalization of these four levels. Based on the detail provided in the studies we assessed, the distinction between ‘communication’ and ‘consultation’ was unclear. Similarly, the details defining ‘collaboration’ from ‘coproduction’ were missing in published reports. Therefore, we have opted to simplify this by categorizing engagement into two levels. Our first level is ‘advice/feedback’ which includes both the communication and consultation levels. Stakeholder opinions, perspectives, experiences, or values are sought and considered by the guideline development team. Our second level of engagement is ‘decision-making’ which includes collaboration and co-production; stakeholders actively contribute to making decisions at the different stages of the guideline’s development and recommendations.

While there are nuances within each of our two levels of engagement, we have decided that the use of two clearly distinct levels is more feasible for the assessment of stakeholder engagement and also simplifies the guidance we will include in our planned extension of the GIN-McMaster Checklist.

### Evaluation of engagement

Engagement can improve the usefulness of guidelines and recommendations and increase uptake. Engagement works best when it is multi-directional [5], meaning that all stakeholders are engaged with each other as well as the guideline development group. For engagement to be meaningful, there have to be benefits to the guideline being developed and the stakeholders need to feel that they benefited from the development process.

It is important to have a process and tools to evaluate whether the stakeholder engagement goals of guideline developers were met. This requires clarifying the rationale for engaging stakeholders, who was engaged, the engagement activities, as well as how effective the engagement was [17]. It also requires assessing the characteristics of the engagement and whether feedback was accounted for, and whether contributions to decision making were effective. We plan to develop tools to evaluate stakeholder engagement and use them to evaluate our own attempt at stakeholder engagement throughout this project. We will build on existing tools for evaluating stakeholder engagement in research, such as the Patients Active in Research and Dialogues for an Improved Generation of Medicines (PARADIGM) Patient Engagement Monitoring and Evaluation Framework (https://onlinelibrary.wiley.com/doi/10.1111/hex.13191), and the Patient Engagement in Research Scale (PEIRS) (https://onlinelibrary.wiley.com/doi/full/10.1111/hex.13227). We also plan to produce guidance for using these processes and tools to evaluate stakeholder engagement in guideline development.

### Documentation and transparency

Including multiple stakeholders in the guideline development process will necessitate an increased attention to transparency around the methods of engagement as well as potential conflicts of interest (see next section). Recommendations for transparently reporting guidelines already exist [18]. The RIGHT (Reporting Items for practice Guidelines in HealThcare) checklist includes 22 items related to reporting of basic information, background, evidence, recommendations, review and quality assurance, funding, and declaration and management of interests. There are two items relevant to reporting of stakeholder engagement; reporting how all contributors were selected and their roles and responsibilities as well as describing how conflicts of interest were evaluated and managed.

We plan to develop an extension of the RIGHT checklist to include additional items related to engaging different stakeholder groups to address this gap and encourage complete and transparent reporting.

### Conflict of interest management

**Fig. 1:**
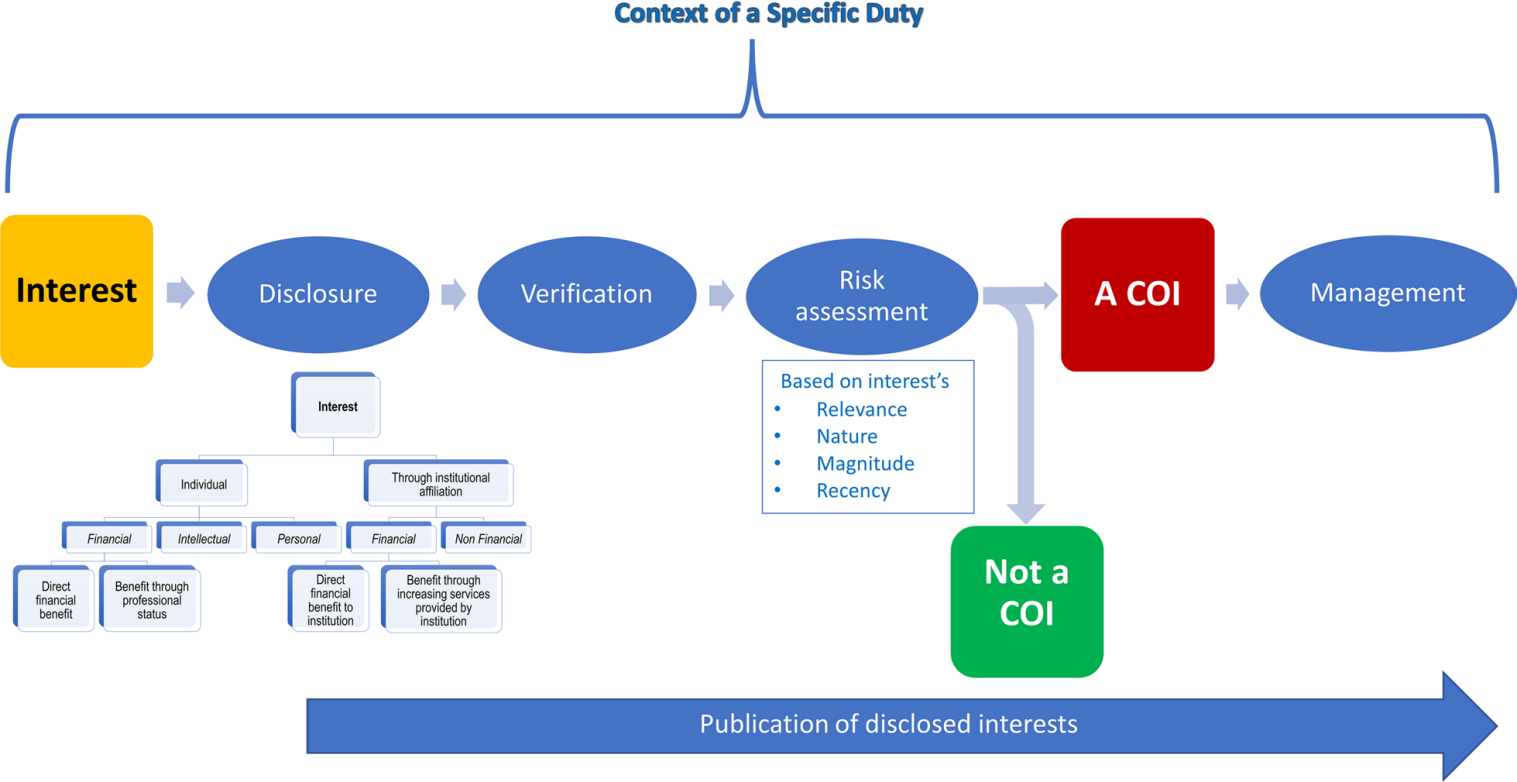
Categorization of interests and their risk assessment in health research in the context of conflict-of-interest policies [11]

While the goal of multi-stakeholder engagement is to bring a multitude of views and perspectives to the table, it can bring additional conflicts of interest. A conflict of interest exists when “a past, current or future interest creates a risk of inappropriately influencing an individual’s judgment, decision, or action when carrying out a specific duty”[11] (Fig. 1). The duty of a stakeholder representative in the context of guideline development is to develop recommendations that guide clinical treatment decisions and safeguard the interests of the groups the recommendations are intended to serve (typically patients). It is important to distinguish between stakeholder representatives’ conflicts of interests and their ‘legitimate interests’. The latter refer to the interests of the stakeholder group that the individual is representing (e.g., ensuring the recommendation reflects the values and preferences of patients). Those interests should not be used to restrict the contribution of the stakeholder representatives to the guideline development process.

A recently published framework categorizes conflicts of interest according to their level (personal or institutional) and their type (financial, intellectual, personal, and cultural). These conflicts might vary across the different stakeholders. For example, principal investigators could be intellectually conflicted if their research is relevant to specific recommendations. Patient advocates might have institutional conflicts of interest emanating from their organizations’ interests [19]. The same applies to providers who are members of professional medical associations [20].

Once stakeholder representatives disclose their interests, the guideline developing organization may assess whether the risk associated with each disclosed interest qualifies as a conflict (based on the relevance, nature, magnitude, and recency of the interest). The final step is to manage any conflicts of interests. Such management should strike a balance between ensuring representativeness of the different stakeholders while minimizing bias. While guidance for conflict of interest management in guideline development is available [21, 22], guidance that specifically addresses the management of conflict of interest in the context of multi-stakeholder engagement is lacking. We plan to fill this gap by producing guidance around managing stakeholder conflicts of interest in guideline development.

## Discussion

To address all the considerations identified in our discussions with stakeholders, we have planned for a series of papers. We will develop guidance for managing conflicts of interest across multiple stakeholders in guideline development; a reporting guideline to encourage transparent reporting of stakeholder engagement in guidelines; and finally, we will describe the methods we have used throughout this project to engage with our own stakeholders, reporting on the barriers and facilitators as well as lessons learned.

Our overall goal is to develop a GIN-McMaster Guideline Development Checklist Extension for Stakeholder Engagement. Our previous meetings informed the development of an international survey to obtain broad opinions about the engagement of each stakeholder group in the stages of guideline development and we gathered in depth insight into the engagement of each group through interviews. These will help us contextualize the feedback we have received and to finalize the checklist extension. We have already published a list of criteria for selecting individuals to represent identified stakeholder groups [12].

Our planned checklist extension will allow for variability in views and flexibility for different settings and guideline contexts and resources. The guidance will focus on which stages of guideline development each stakeholder group should be engaged in; whether the engagement should be in a decision-making or feedback role; and the optimal engagement of stakeholder across the different stages of guideline development. Guidance for engaging with stakeholders, especially patients, or providers [26, 27] as well as other stakeholder groups such as product makers exists related to research and medical product development [28]. However, to our knowledge, our project is the first to develop guidance for engaging with multiple stakeholder groups throughout health and healthcare guideline development.

The work of MuSE and the guidance produced may be generalizable or adaptable for stakeholder engagement in other areas, such as health technology assessment (HTA) and systematic reviews.

## Data Availability

Not applicable.

## Appendix 1

### Participants

**Table:**

	Name	Country	Stakeholder Group
1	Alba Antequera	Spain	PI
2	Ana Marusic	Croatia	Peer Review Editor
3	Angus Gunn	UK	Product Maker
4	Asma Ben Brahem	Tunisia	Policymaker
5	Marc Avey	Canada	Program Manager
6	Behrang Kianzad	Denmark	Payer/Purchaser of health services
7	Bev Shea	Canada	PI
8	Christine Laine	USA	Peer Review Editor
9	Elizabeth Ghogomu	Canada	PI
10	Comfort Ekanem	Nigeria	Providers
11	Thomas Concannon	USA	PI
12	Diana Ingram	USA	Provider
13	Soumyadeep Bhaumik	India	Peer Review Editor
14	Elie Akl	Lebanon	PI
15	Eddy Lang	Canada	PI
16	Elena Parmelli	Belgium	PI
17	Emily Cahill	USA	Program Manager
18	Eve Tomlinson	UK	PI
19	Hussain Jafri	Pakistan	Patient
20	Imad Bou Akl	Lebanon	Provider
21	Ina Kopp	Germany	PI
22	Jane Cowl	UK	Public
23	Janet Hatcher Roberts	Canada	PI
24	Jennifer Hilgart	UK	PI
25	Jennifer Petkovic	Canada	PI
26	Joanne Khabsa	Lebanon	PI
27	Jordi Pardo Pardo	Canada	PI
28	Karen Head	UK	PI
29	Kevin Pottie	Canada	PI
30	Tanja Kuchenmüller	Germany	Policymaker
31	Lara Maxwell	Canada	PI
32	Laura Dormer	UK	Peer Review Editor
33	Ligia Teixeira	UK	Program Manager
34	Lorenzo Moja	Italy	Payer/Purchaser of health services
35	Lyubov Lytvyn	Canada	Canada
36	Marisha Palm	USA	Public
37	Maureen Smith	Canada	Patient
38	Lawrence Mbuagbaw	Canada	PI
39	Michael Saginur	Canada	Provider
40	Navin Sewak	UK	Product Maker
41	Nevilene Slingers	South Africa	Program Manager
42	Olivia Magwood	Canada	PI
43	Parker Roses	UK	PI
44	Pearl Atwere	Canada	PI
45	Peter Tugwell	Canada	PI
46	Alex Todhunter-Brown	UK	PI
47	Regina Greer-Smith	USA	Public
48	Richard Morley	UK	Patient
49	Rosiane Simeon	Canada	PI
50	Sally Crowe	UK	Public
51	Holger Schünemann	Canada	PI
52	Sophie Staniszewska	UK	PI
53	Sophie Glatt	UK	Product Maker
54	Tamara Kredo	South Africa	PI
55	Tamara Lotfi	Canada	PI
56	Thurayya Arayssi	Qatar	PI
57	Vivian Welch	Canada	PI
58	Wojtek Wiercioch	Canada	PI

## Appendix 2

### GRIPP2 checklist

**Table:**

Section and topic	Item	Reported on page No
Section 1: Abstract of paper		
1a: Aim	Report the aim of the study	3
1b: Methods	Describe the methods used by which patients and the public were involved	3
1c: Results	Report the impacts and outcomes of PPI in the study	3
1d:Conclusions	Summarise the main conclusions of the study	3
1e: Keywords	Include PPI, “patient and public involvement,” or alternative terms as keywords	3
Secion 2: Background to paper		
2a: Definition	Report the definition of PPI used in the study and how it links to comparable studies	4
2b: Theoretical underpinnings	Report the theoretical rationale and any theoretical influences relating to PPI in the study	N/A
2c: Concepts and theory development	Report any conceptual or theoretical models, or influences, used in the study	N/A
Section 3: Aims of paper		
3: Aim	Report the aim of the study	4
Section 4: Methods of paper		
4a: Design	Provide a clear description of methods by which patients and the public were involved	7
4b: People involved	Provide a description of patients, carers, and the public involved with the PPI activity in the study	5, 7
4c: Stages of involvement	Report on how PPI is used at different stages of the study	7
4d: Level or nature of involvement	Report the level or nature of PPI used at various stages of the study	7
Section 5: Capture or measurement of PPI impact		
5a: Qualitative evidence of impact	If applicable, report the methods used to qualitatively explore the impact of PPI in the study	N/A
5b: Quantitative evidence of impact	If applicable, report the methods used to quantitatively measure or assess the impact of PPI	N/A
5c: Robustness of measure	If applicable, report the rigour of the method used to capture or measure the impact of PPI	N/A
Section 6: Economic assessment		
6: Economic assessment	If applicable, report the method used for an economic assessment of PPI	N/A
Section 7: Study results		
7a: Outcomes of PPI	Report the results of PPI in the study, including both positive and negative outcomes	7–12
7b: Impacts of PPI	Report the positive and negative impacts that PPI has had on the research, the individuals involved (including patients and researchers), and wider impacts	N/A
7c: Context of PPI	Report the influence of any contextual factors that enabled or hindered the process or impact of PPI	N/A
7d: Process of PPI	Report the influence of any process factors, that enabled or hindered the impact of PPI	N/A
7ei: Theory development	Report any conceptual or theoretical development in PPI that have emerged	N/A
7eii: Theory development	Report evaluation of theoretical models, if any	N/A
7f: Measurement	If applicable, report all aspects of instrument development and testing (eg, validity, reliability, feasibility, acceptability, responsiveness, interpretability, appropriateness, precision)	N/A
7 g: Economic assessment	Report any information on the costs or benefit of PPI	N/A
Section 8: Discussion and conclusions		
8a: Outcomes	Comment on how PPI influenced the study overall. Describe positive and negative effects	N/A
8b: Impacts	Comment on the different impacts of PPI identified in this study and how they contribute to new knowledge	N/A
8c: Definition	Comment on the definition of PPI used (reported in the Background section) and whether or not you would suggest any changes	N/A
8d: Theoretical underpinnings	Comment on any way your study adds to the theoretical development of PPI	13
8e: Context	Comment on how context factors influenced PPI in the study	N/A
8f: Process	Comment on how process factors influenced PPI in the study	N/A
8 g: Measurement and capture of PPI impact	If applicable, comment on how well PPI impact was evaluated or measured in the study	10
8 h: Economic assessment	If applicable, discuss any aspects of the economic cost or benefit of PPI, particularly any suggestions for future economic modelling	N/A
8i: Reflections/critical perspective	Comment critically on the study, reflecting on the things that went well and those that did not, so that others can learn from this study	N/A
